# Buried-Gate Flexible CNT FET with HZO Dielectric on Mica Substrate

**DOI:** 10.3390/nano15161218

**Published:** 2025-08-09

**Authors:** Haiou Li, Jiamin Shen, Zhihao Zhuo, Fabi Zhang, Xingpeng Liu, Qing Liao

**Affiliations:** Guangxi Key Laboratory of Precision Navigation Technology and Application, Guilin University of Electronic Technology, Guilin 541004, China; lihaiou@guet.edu.cn (H.L.); shenjiammin@gmail.com (J.S.); willcy_722@hotmail.com (Z.Z.); zhangfabi@outlook.com (F.Z.)

**Keywords:** carbon nanotubes, flexible electronics, mica, thin film transistors (TFTs), interface planarization

## Abstract

Carbon nanotube field-effect transistors (CNT FETs) are considered strong candidates for next-generation flexible electronics due to their excellent carrier mobility and mechanical flexibility. However, the fabrication of CNT FETs on conventional flexible substrates such as PI or PET is often limited by surface roughness, chemical incompatibility, and poor mechanical robustness, resulting in degraded device performance. In this study, we report the fabrication of buried-gate CNT FETs incorporating Hf_0.5_Zr_0.5_O_2_ as the gate dielectric on mica substrates, which offer high surface flatness, low defect density, and superior mechanical durability. The fabricated devices exhibit outstanding electrical characteristics, including a field-effect mobility of 38.4 cm^2^/V·s, a subthreshold swing of 93 mV/dec, and a transconductance of 14.2 μS. These results demonstrate the excellent mechanical stability and reliable electrical performance of the proposed devices under bending stress, highlighting their suitability for mechanically demanding flexible electronics applications.

## 1. Introduction

Flexible electronic devices are an emerging field of study in the twenty-first century, with great potential for usage in biological sensors, artificial synapses, and flexible integrated circuits [1,2,3,4]. Carbon nanotubes have a wide range of potential applications due to their outstanding mechanical properties, such as high strength and flexibility. They can also be applied to any flexible substrate, which allows them to survive mechanical deformation [5,6]. However, CNT FETs perform significantly differently on rigid and flexible silicon substrates, and the development of flexible CNT FETs faces major technical challenges. Conventional polymer substrates, such as polyimide (PI) and polyethylene terephthalate (PET), are widely used due to their transparency and flexibility; however, their poor temperature resistance and high coefficient of thermal expansion severely limit high-temperature process compatibility and reduce device performance. Excellent flexibility and stable electrical properties with no degradation after deformation are essential for flexible device applications. According to research, selecting or refining substrates with exceptional electrical properties can help boost transistor performance even more [7].

In this work, we used natural fluorine gold mica as a substrate, which has excellent temperature stability and mechanical durability [8,9]. Mica has exceptional mechanical properties, stability, and resistance to mechanical fatigue at bending radii of less than 2.5 mm, even after 1000 consecutive bends [10]. Mica’s surface is atomically smooth, with no grain boundary imperfections after stripping. This natural surface with low roughness (RMS < 0.2 nm) is perfect for manufacturing ultra-thin dielectric layers [11,12]. The roughness of the substrate significantly affects the uniformity of deposited hafnium oxide films. This, in turn, affects the device’s reliability [13]. Achieving highly uniform film deposition would further improve the performance of carbon nanotube field-effect transistors (FETs) [14,15]. In this study, mica exfoliation was performed manually to preserve surface integrity. Nevertheless, scalable techniques such as roll-to-roll (R2R) delamination and automated layer transfer processes are currently being developed and may be adopted in future work to enable large-area integration of flexible devices [16].

HfO_2_ is widely used as a high-k gate dielectric in advanced microelectronic devices due to its high dielectric constant, thermal stability, and compatibility with CMOS techniques [17]. However, undoped HfO_2_ forms stable monoclinic phases at ultrathin scales, without spontaneous polarization, which limits its future use in ferroelectric field-effect transistors (FeFETs) [18]. To overcome this limitation, the materials’ phase stability and polarization properties were greatly increased by adding zirconium (Zr) to form Zr-doped hafnium zirconium oxide (Hf_0.5_Zr_0.5_O_2_) [19]. Zr doping efficiently prevents the formation of the monoclinic phase [20], promoting the stable existence of non-centrosymmetric orthorhombic phases, resulting in a lower leakage current density and higher electrical reliability, as well as a more controllable oxygen vacancy concentration in HZO. This makes it suitable for use in high-speed, low-power devices [21]. Notably, both HfO_2_ and HZO in this study were deposited using atomic layer deposition (ALD), a technique known for producing uniform, conformal, and pinhole-free dielectric films. Compared with HfO_2_, ALD-grown HZO not only achieves a higher dielectric constant but also exhibits enhanced polarization due to its ferroelectric nature and improved grain boundary alignment during crystallization.

Furthermore, standard device techniques confront two core challenges when producing flexible CNT FETs: the complexity of the transfer process and the reliability of device lift-off. There are typically two methods: one is to fabricate CNT FETs or deposit CNT films on rigid silicon substrates and then transfer them to flexible substrates (e.g., PET/PI) via wet transfer [22]. During the device transfer process, the lift-off step faces multiple challenges: Polymer residues can degrade contact interfaces; HF etching of the SiO_2_ sacrificial layer requires precise control to prevent film damage or contamination. The spin-coating and removal of PMMA support layers may also introduce mechanical defects such as wrinkles or fractures. Furthermore, direct separation of flexible devices often results in failure due to abrupt changes in interface roughness or metal layer cracking. While sacrificial layers can buffer mechanical stress, the prolonged, strong acid/alkali etching process may corrode functional layers and reduce carrier mobility, ultimately leading to performance degradation. The other is to fabricate CNT FETs directly on PET/PI flexible substrates, but this method has strict temperature requirements during fabrication and must be kept completely at low temperatures [23].

Therefore, by fabricating devices directly on rigid mica and then mechanically stripping them off, we can control the substrate thickness freely, enabling fast, low-cost, and damage-free layering [24]. This approach simplifies the manufacturing process, enhances overall device performance and stability, and reduces transfer process damage and flaws. This significantly improves the device’s reliability. It finds a compromise between rigidity and flexibility in the pursuit of high-performance and flexible devices. The use of mica as a flexible CNT FET substrate has the advantage of combining inorganic material performance with flexible substrate adaptability. It is ideal for flexible electronic devices that require high-quality connections, electrical stability, and heat treatment, and it outperforms ordinary polymer substrates in this regard. This paper constructs a flexible CNT FET device with a buried-gate structure based on HZO gate dielectric, investigates its electrical stability under high bending stress conditions, and demonstrates its potential application in new-generation flexible smart electronic systems.

## 2. Materials and Methods

Fabrication of flexible CNT FETs. First, the 1 cm × 1 cm mica substrates were cleaned with acetone, alcohol, and deionized water for 15 min in an ultrasonic machine and then blow-dried with nitrogen. Next, for deep cleaning, any remaining organic material was eliminated by an oxygen plasma machine. Photolithography was used to pattern the gate region, followed by RIE (CF_4_:O_2_ = 3:1, power: 100 W, 50 s) to etch a 50 nm-deep gate trench. Then, 50 nm of Au was deposited by thermal evaporation to form the gate electrode. A 10 nm HfO_2_ gate dielectric layer was deposited by ALD, offering excellent surface flatness and thermal stability. Next, source and drain electrodes were patterned by photolithography, and 100 nm of Au was deposited by thermal evaporation. Subsequently, a CNT thin film was deposited. The fabricated CNT FET featured a channel length and width of 5 μm and 40 μm, respectively. The CNTs outside the channel were removed by RIE (O_2_, power: 80 W, 30 s) to prevent short circuits.

Preparation of CNT thin films. This experiment used a 99.99% CNT dispersion to prepare thin films by dip-coating. To prevent nanotube aggregation, the CNT solution was first sonicated for 1 min using a low-power ultrasonic cleaner. The fabricated devices were then immersed in the dispersion for 24 h to enable uniform CNT film formation. After forming a uniform CNT film, the device was vertically withdrawn at a low speed to prevent aggregation. It was then immersed in toluene for 10 min and gently rinsed with isopropanol and deionized water to remove organic residues. Finally, baking at 120 °C for 10 min eliminated residual solvents and improved film density, enhancing both electrical stability and mechanical durability.

Delamination and Bending of CNT TFTs. By mechanical exfoliation, the rigid mica substrate can be thinned down to an ultrathin layer of 1–10 μm, exhibiting flexibility with a bending radius of less than 3.5 mm. After delamination, CNT FETs on mica were mounted onto bending molds with radii of 3.5 mm and 6 mm for mechanical testing.

## 3. Results

**Figure 1:**
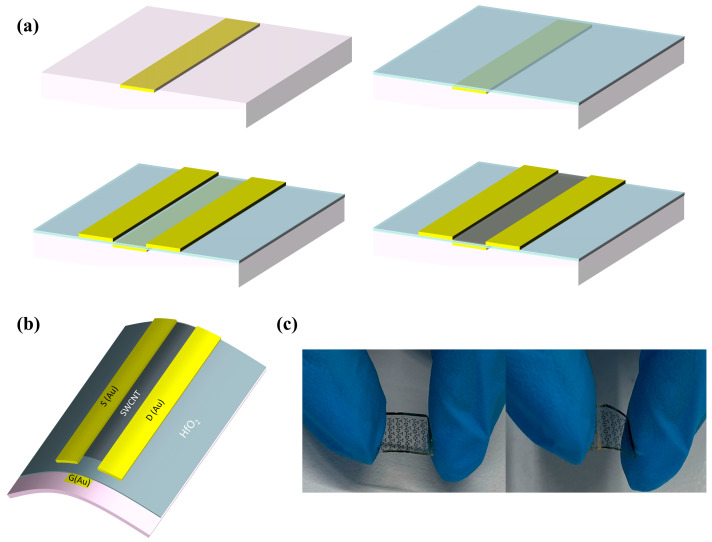
Schematic illustration of the flexible device. (**a**) Fabrication process of self-aligned buried-gate CNT FET. (**b**) Schematic diagram of curved structure. (**c**) Optical images of the device, relaxed and strained.

Figure 1a shows the preparation process and structure of self-aligned CNT FETs. Top-gate CNT FETs suffer from poor dielectric deposition due to the lack of dangling bonds on CNT surfaces, leading to non-uniform HfO_2_ coverage and compromised gate control. To address this, a buried-gate architecture was employed, allowing the dielectric to be deposited on metal, thus improving interface quality and film uniformity [25]. In this study, a buried-gate structure was adopted to enhance gate control, improve interface quality, reduce power consumption, and achieve better film uniformity. By placing the CNT channel on the top layer, the design protects it from physical damage during subsequent metal deposition and isolates it from environmental factors such as humidity, thereby improving long-term device reliability [26,27]. Figure 1b shows a schematic diagram of the curved structure. Figure 1c depicts the physical appearance of the fabricated device in its flat and bent states, respectively, which confirms its inherent mechanical flexibility.

**Figure 2:**
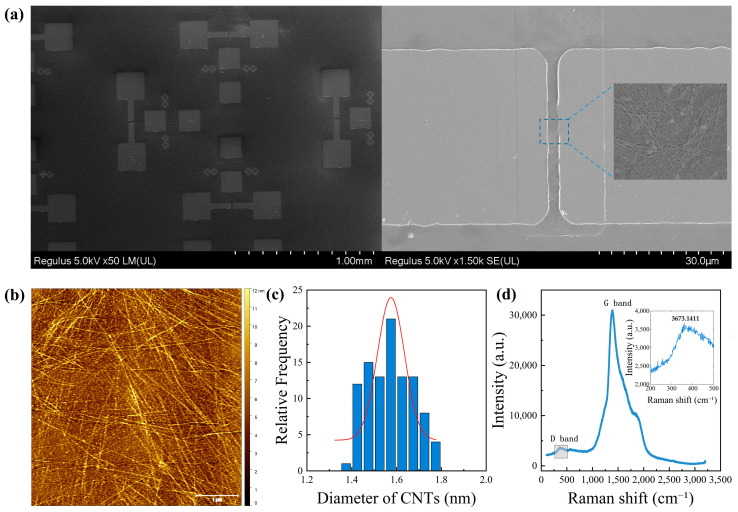
(**a**) SEM images of the CNT FET array and CNT distribution in the channel. (**b**) AFM image of carbon nanotubes in the channel region. (**c**) Diameter distribution of 100 CNTs measured by SEM. (**d**) Raman spectroscopy of the CNT film.

The quality of SWCNTs employed in CNT FETs is a critical factor influencing both research outcomes and practical applications. Accordingly, Figure 2a,b present AFM and SEM images of CNTs within the device channel. A statistical analysis of 100 CNTs (Figure 2c) demonstrates a narrow diameter distribution between 1.4 and 1.7 nm, indicative of high structural order and crystallinity. This uniformity minimizes the occurrence of multi-walled structures and defects, thereby contributing to improved device-to-device performance consistency in CNT-based electronics [28]. Figure 2d presents the Raman spectrum of the fabricated CNT sample. The G band appears at approximately 1590 cm^−1^ with an intensity of 30,997.77 a.u., corresponding to the in-plane vibrational mode of sp^2^-hybridized carbon atoms, indicating a well-graphitized structure. The D band, located around 1350 cm^−1^ with an intensity of 3673.14 a.u., reflects the presence of minor structural defects. The intensity ratio of the G to D bands is approximately 8.44, suggesting a low defect density and high crystallinity of the CNTs. The consistent Raman features and diameter distribution demonstrate the high structural purity and well-controlled synthesis of the carbon nanotubes, making them suitable for high-performance electronic devices.

**Figure 3:**
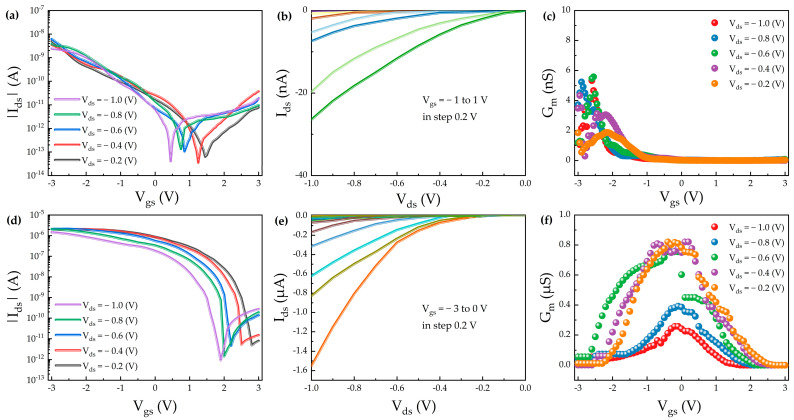
HfO_2_ gated carbon nanotube field effect transistor. (**a**) Transfer characteristics of back-gate CNT FET. (**b**) Output characteristics (back-gate) (**c**) Transconductance (back-gate). (**d**) Transfer characteristics of buried-gate CNT FET. (**e**) Output characteristics (buried-gate). (**f**) Transconductance (buried-gate).

In this study, 50 nm of gold (Au) was employed as the gate contact electrode due to its high work function and excellent chemical stability. Two types of CNT FETs were fabricated: a conventional back-gate structure and a buried-gate structure. Both devices utilized a 10 nm-thick HfO_2_ gate dielectric layer deposited via atomic layer deposition (ALD), and 100 nm of Au was used as the source/drain contact electrodes. Electrical characterizations were conducted at room temperature using a Keithley 2636b semiconductor parameter analyzer. Transfer characteristics were measured by sweeping the gate voltage (V_gs_) from −3 V to +3 V under various drain-source voltages (V_ds_) ranging from −0.2 V to −1 V. The measured transfer curves primarily exhibited p-type behavior, accompanied by a minor n-type component. This ambipolar behavior is commonly observed in CNT FETs due to the small bandgap of the carbon nanotubes [29,30]. Figure 3a–c present the electrical characteristics of the back-gate CNT FET. The transfer and output characteristics of the buried-gate CNT FET are shown in Figure 3d and Figure 3e, respectively, while Figure 3f illustrates the extracted transconductance (G_m_). When V_ds_ was increased from −0.2 V to −1 V, the buried-gate CNT FET exhibited a stable on/off current ratio in the range of 10^5^ to 10^6^. The transconductance increased from 0.25 μS to 0.82 μS, with a threshold voltage of approximately 0.5 V. The field-effect mobility, calculated from the linear regime of the transfer characteristics, reached 14.47 cm^2^/V·s.

Compared with the back-gate structure, the buried-gate CNT FET demonstrated significant performance advantages. The on/off ratio improved by 2–3 orders of magnitude, and the carrier mobility increased by approximately 80%. Furthermore, the subthreshold swing (SS) was reduced from 330 mV/dec to 105 mV/dec, and the transconductance increased from 0.6 μS to 0.82 μS, indicating enhanced interface quality and electrical performance. The superior performance of the buried-gate structure can be attributed to the enhanced gate control over the channel. This architecture effectively reduces the source/drain barrier height and suppresses edge effects, leading to higher carrier concentration and improved channel conductivity. As a result, the on-state current (I_on_) increased significantly from 6 × 10^−9^ A (back-gate) to 2 × 10^−6^ A (buried-gate), highlighting the improved electrical tunability and conductive performance of the buried-gate CNT FET. Moreover, the improved controllability of the gate/channel interface leads to a reduction in interface trap density (D_it_), which is beneficial for enhancing both the carrier mobility and the on/off current ratio of the device. It is well established that the SS serves as a key parameter for quantitatively evaluating D_it_. Under the assumption of a uniform trap distribution, the interface trap density in the channel can be approximated by the following expression:

(1) $$D_{it}=\frac{C_{ox}}{q}\cdot \left(\frac{SS}{\ln10\cdot \frac{kT}{q}}-1\right)$$

According to Equation (1), C_ox_ denotes the gate oxide capacitance per unit area, q is the elementary charge, k is the Boltzmann constant, and T is the absolute temperature. Based on the above equation, the interface trap density D_it_ of the buried-gate CNT FET is calculated to be 3.91 × 10^12^ cm^−2^·eV^−1^. The interface trap density is a critical metric for evaluating the interfacial quality between the gate dielectric and the channel material [31]. The obtained value indicates a relatively low density of interface states, suggesting that the device exhibits excellent gate electrostatic control and a reduced subthreshold swing. This result confirms that effective interface engineering has been achieved at the HfO_2_/CNT interface [32]. Overall, the buried-gate architecture not only offers superior electrical performance but also provides enhanced process compatibility compared to the conventional back-gate structure. These advantages make it a promising platform for the development of high-performance, highly integrated CNT FETs.

**Figure 4:**
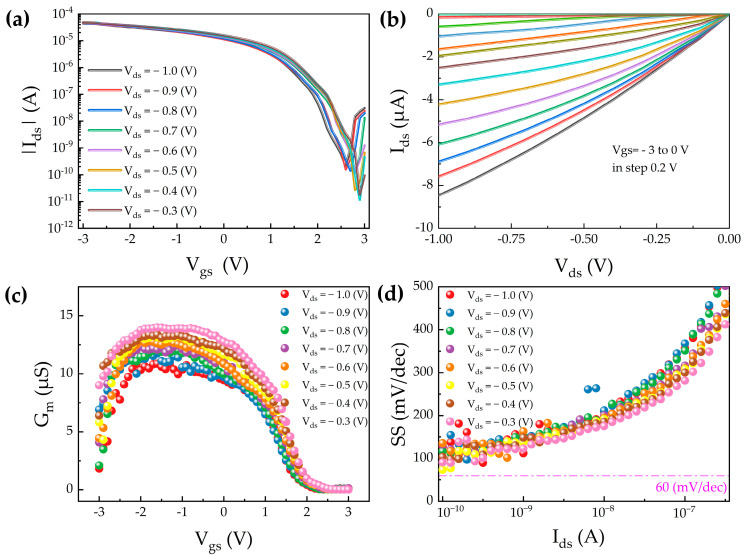
Buried-gate carbon nanotube field-effect transistor with Hf_0.5_Zr_0.5_O_2_ gate dielectric. (**a**) Transfer characteristics. (**b**) Output characteristics. (**c**) Transconductance. (**d**) Subthreshold swing.

In carbon nanotube field-effect transistors, the thermal stability and mechanical reliability of the gate dielectric material are critical factors influencing overall device performance. Although conventional hafnium oxide (HfO_2_) exhibits excellent CMOS compatibility and favorable dielectric properties, it suffers from phase instability under high-temperature or mechanically flexible conditions. Specifically, HfO_2_ tends to transition from a metastable orthorhombic phase to a stable monoclinic phase, leading to degradation in ferroelectricity and high-k characteristics. This structural transition can significantly impair SS and drive current in CNT FETs. To address these limitations, a doped variant, Hf_0.5_Zr_0.5_O_2_ (HZO), obtained by alloying zirconium oxide (ZrO_2_) and HfO_2_ in a 1:1 ratio, has emerged as a promising alternative. HZO exhibits a higher dielectric constant and stronger gate electrostatic control, thereby enabling the realization of high-performance flexible CNT FETs. Importantly, HZO stabilizes the non-centrosymmetric orthorhombic ferroelectric Pca2_1_ phase via zirconium incorporation, offering enhanced stress resistance and thermal retention [33]. This stability is maintained even at ultrathin thicknesses, resulting in favorable low-voltage operation characteristics [34]. To induce and preserve the orthorhombic ferroelectric phase with robust performance, post-deposition annealing at elevated temperatures is essential for Hf–Zr–O based ferroelectric thin films. Consequently, the choice of flexible substrate must also accommodate high thermal budgets. Compared to common polymer substrates such as polyimide (PI) and polyethylene terephthalate (PET), mica substrates offer superior thermal stability, making them an ideal platform for flexible electronics applications [35]. Based on the above material considerations and structural design, we developed buried-gate flexible CNT FETs incorporating HZO as the gate dielectric. The performance of these devices is systematically compared with that of buried-gate CNT FETs based on conventional HfO_2_ dielectrics to elucidate the advantages of the HZO integration strategy. Figure 4a,b present the transfer and output characteristics of the flexible CNT FET based on the buried-gate structure with HZO as the gate dielectric. The device exhibits typical p-type behavior. The transfer characteristics demonstrate a clear distinction between the on- and off-states, achieving an on/off current ratio exceeding 10^6^, indicative of excellent switching behavior and strong gate modulation capability. The output characteristics show good linear and saturation regions, with a significant increase in drain current as the gate voltage increases, reflecting superior carrier transport and gate control. As shown in Figure 4c, the G_m_ reaches 14.2 μS. Figure 4d shows that the SS is as low as 92 mV/dec, and the field-effect mobility is extracted to be 38.4 cm^2^/V·s. These results confirm the high electrical performance of the HZO-based device.

**Figure 5:**
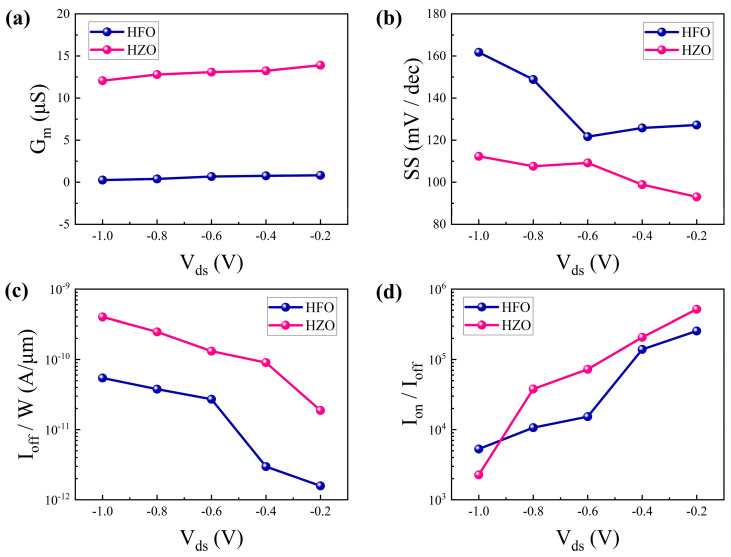
Performance comparison between HfO_2_ and Hf_0.5_Zr_0.5_O_2_ buried-gate CNT FETs: (**a**) G_m_. (**b**) SS. (**c**) I_off_/W. (**d**) I_on_/I_off_.

To further evaluate the impact of different gate dielectrics, Figure 5a–d compare the electrical performance of CNT FETs using HZO and HfO_2_ dielectrics. As shown in Figure 5a, when V_ds_ increases from 0.2 V to 1 V, the transconductance of the HZO-based device remains around 14 μS, significantly higher than the 0.8 μS observed in the HfO_2_-based device, indicating enhanced amplification and gate control capability. Figure 5b reveals that at V_gs_ = −0.2 V, the SS of the HZO device is 92 mV/dec, lower than the 105 mV/dec of the HfO_2_ device, implying reduced power consumption. Furthermore, as shown in Figure 5c, both devices exhibit low normalized off-state current (I_off_/W), ensuring minimal leakage in the off-state. Figure 5d demonstrates the I_on_/I_off_ ratios of the two devices, where the HZO-based FET achieves 2.7 × 10^6^, outperforming the 2.5 × 10^5^ of the HfO_2_-based device, thus confirming superior switching characteristics. In summary, the flexible CNT FET employing HZO as the high-k gate dielectric exhibits enhanced performance in terms of transconductance, subthreshold swing, on/off ratio, and mobility, highlighting its potential for high-performance flexible electronic applications.

**Figure 6:**
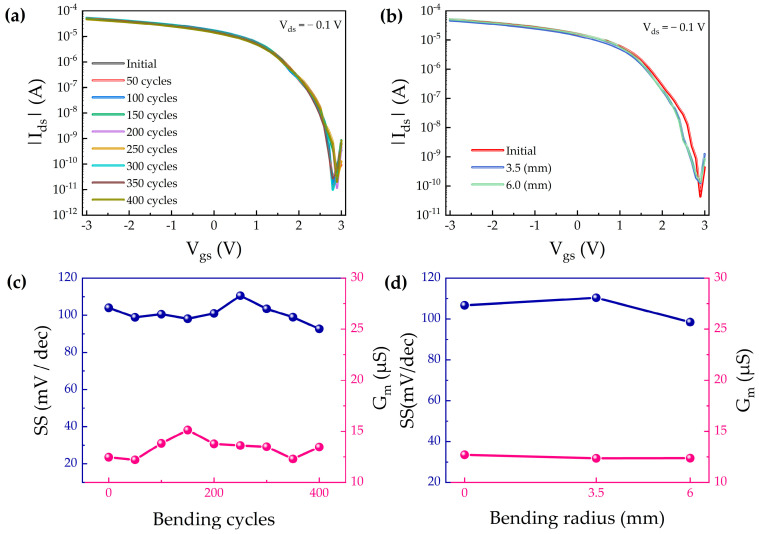
Bending test of flexible Hf_0.5_Zr_0.5_O_2_ devices. (**a**) Transfer characteristics of the device after 400 bending cycles at V_ds_ = − 0.1 V. (**b**) Transfer characteristics of the device at V_ds_ = − 0.1 V under bending radii of 3.5 mm and 6 mm. Transconductance and subthreshold swing of devices under (**c**) different bending cycles and (**d**) different bending radii.

Bending fatigue testing is a critical evaluation method closely associated with the operational reliability and service life of flexible electronic devices. Previous studies have demonstrated that Hf_0.5_Zr_0.5_O_2_ thin films maintain excellent mechanical flexibility and stability even under a bending radius of 5 mm [36]. To further assess the device’s mechanical endurance under cyclic stress, the CNT FET was subjected to 400 cycles of repeated bending using a 3.5 mm radius mold, and the corresponding transfer characteristics were recorded at various bending cycles, as shown in Figure 6a. The results indicate that the device retains stable electrical performance throughout the fatigue test. In addition, to evaluate the device’s robustness under varying mechanical strain levels, Figure 6b presents the transfer curves obtained under different bending radii of 6 mm and 3.5 mm. To quantify the impact of bending-induced stress, electrical performance was further assessed through bias stress tests and key parameter extraction, as illustrated in Figure 6c,d. Notably, even after 400 bending cycles, both the SS and G_m_ exhibited variations of less than 10%, demonstrating excellent mechanical durability and stable gate modulation under deformation. Overall, the device exhibits outstanding environmental stability and mechanical resilience during bending tests, indicating its suitability for next generation flexible and stretchable electronics intended for demanding applicationscenarios.

## 4. Conclusions

In this study, we have fabricated high-performance flexible CNT FETs based on a buried-gate structure using Hf_0.5_Zr_0.5_O_2_ as the gate dielectric and mica as the substrate. The addition of Zr stabilizes the orthorhombic ferroelectric phase in HZO, enhancing gate control and electrical performance. The fabricated devices exhibit a high field-effect mobility of 38.4 cm^2^/V·s, a low subthreshold swing of 93 mV/dec, and a transconductance of 14.2 μS, with a low interface trap density of 3.91 × 10^12^ cm^−2^·eV^−1^, indicating excellent dielectric/channel interface quality. Mechanical bending tests with radii of 6 mm and 3.5 mm over 400 cycles confirm that key parameters, including SS and Gm, remain within 10% variation, demonstrating outstanding mechanical reliability. These results underscore the synergistic advantages of the buried-gate structure, high-k ferroelectric dielectric, and flexible mica substrate, positioning this device as a promising candidate for next-generation flexible logic circuits, wearable biomedical sensors, and neuromorphic electronics. Future work will incorporate TEM and AFM characterization to explore interfacial and morphological evolution under mechanical stress, further advancing the understanding of device reliability. While HfO_2_ remains the industry-standard high-k dielectric in CMOS nodes due to its excellent reliability, simpler process control, and integration maturity, our findings demonstrate that, within the specific context of flexible electronics, buried-gate architectures, and mica substrates, HZO offers practical advantages worth further exploration. Although dedicated C–V hysteresis measurements were not performed in this work, our focus being primarily on transfer characteristics and mechanical reliability, forward I_d_–V_g_ sweeps exhibited negligible hysteresis, indicating stable switching behavior of the HZO-based CNT FETs. A more detailed C–V hysteresis study will be pursued in future investigations to provide deeper insight into charge trapping and interface properties.

## Data Availability

The data presented in this study are available on request from the corresponding author due to privacy considerations.
